# Estimation of Citywide Air Pollution in Beijing

**DOI:** 10.1371/journal.pone.0053400

**Published:** 2013-01-08

**Authors:** Jin-Feng Wang, Mao-Gui Hu, Cheng-Dong Xu, George Christakos, Yu Zhao

**Affiliations:** 1 State Key Laboratory of Resources and Environmental Information System, Institute of Geographic Sciences and Natural Resources Research, Chinese Academy of Sciences, Beijing, China; 2 Department of Geography, San Diego State University, San Diego, California, United States of America; 3 School of Geosciences and Info-Physics, Central South University, Changsha, China; University of Oxford, United Kingdom

## Abstract

There has been discrepancies between the daily air quality reports of the Beijing municipal government, observations recorded at the U.S. Embassy in Beijing, and Beijing residents’ perceptions of air quality. This study estimates Beijing’s daily area PM_2.5_ mass concentration by means of a novel technique SPA (Single Point Areal Estimation) that uses data from the single PM_2.5_ observation station of the U.S Embassy and the 18 PM_10_ observation stations of the Beijing Municipal Environmental Protection Bureau. The proposed technique accounts for empirical relationships between different types of observations, and generates best linear unbiased pollution estimates (in a statistical sense). The technique extends the daily PM_2.5_ mass concentrations obtained at a single station (U.S. Embassy) to a citywide scale using physical relations between pollutant concentrations at the embassy PM_2.5_ monitoring station and at the 18 official PM_10_ stations that are evenly distributed across the city. Insight about the technique’s spatial estimation accuracy (uncertainty) is gained by means of theoretical considerations and numerical validations involving real data. The technique was used to study citywide PM_2.5_ pollution during the 423-day period of interest (May 10, 2010 to December 6, 2011). Finally, a freely downloadable software library is provided that performs all relevant calculations of pollution estimation.

## Introduction

Beijing, the capital city of China, is an international metropolis with a population of over 19 million. As in many big cities worldwide, air pollution is a major concern for city residents. Particulate matter (PM) is the air pollutant that most commonly affects people’s health, where PM_10_ and PM_2.5_ are the two main PM pollutants, i.e., PM consisting of particles with aerodynamic diameters ≤10 µm and ≤2.5 µm, respectively [1], [2]. The sources of PM10 consist of smoke, dirt and dust from factories, farming and roads, as well as mold, spores, and pollen. PM_2.5_ is linked to toxic organic compounds, heavy metals (from smelting, processing, and others), burning of plant material, and forest fires.

PM_2.5_ is a greater health threat than the PM_10_ particles. Laboratory studies have confirmed that the smaller the particle, the more likely it is to lodge in the lungs [3]. In situ studies have shown that these small particles can penetrate indoors, thus altering the home environment. The particles may cause an increase in cardiac and respiratory morbidity and mortality [4]. Indeed, significant increases in deaths from heart and lung disease occur during multi-day periods with high concentrations of fine particles [5]. More than 500,000 deaths per year have been reported worldwide due to PM_2.5_ pollution [6].

In the case of Beijing, there is considerable discrepancy between air pollution levels in terms of PM_10_ records provided by the municipal government, PM_2.5_ observations from individual unofficial stations, and perceptions among the local population. Rapid population growth, urbanization, and greater numbers of vehicles have inevitably caused a considerable increase in air pollution emissions throughout the city [7]–[12]. PM_10_ concentration is a mandatory air quality index that is routinely observed at several official PM_10_ monitoring stations and published daily by the Beijing Municipal Environmental Protection Bureau (BJ-EPB). The U.S. Embassy in Beijing has kept unofficial hourly PM_2.5_ records since spring 2008, using a single monitoring station atop its building [13]. On the other hand, according to BJ-EPB the official stations monitoring Beijing’s air quality are evenly distributed across the city in accordance with relevant scientific standards, whereas the U.S. Embassy data do not accurately represent the overall pollution level in the city [14]. As a result, in the last few years a serious disagreement has emerged between the daily air pollution assessments provided by the BJ-EPB [15], the U.S. Embassy, and those based on population’s perceptions. For example, on October 23, 2011, a thick smog blanket over Beijing revealed a major discrepancy between the categorizations of “slightly polluted” air suggested by BJ-EPB data and “hazardous” air quality determined by U.S. Embassy monitoring [13], [16].

PM_10_ and PM_2.5_ concentrations are related, since most of the PM_10_ is contributed by PM_2.5_ [17]–[19]. Therefore, evaluating the PM_10_-PM_2.5_ relationship can provides information on PM_2.5_ concentrations in areas that are not monitored for it [20], [21]. In this study, we proposed a technique to estimate daily averages of PM_2.5_ concentrations in Beijing, by integrating daily PM_2.5_ observations at the single U.S. Embassy station and their physical correlations with PM_10_ data obtained at a spatially exhaustive monitoring network operated by BJ-EPB. The proposed technique, called SPA (Single Point Areal Estimation), takes advantage of the aforementioned physical link between PM_2.5_ and PM_10_ concentrations to generate areal PM_2.5_ pollution estimates over the entire city. In other words, the PM_10_ observations served as the key secondary information that can improve the estimation of Beijing’s areal daily PM_2.5_ concentration [22].

## Materials and Methods

### Materials

**Figure 1:**
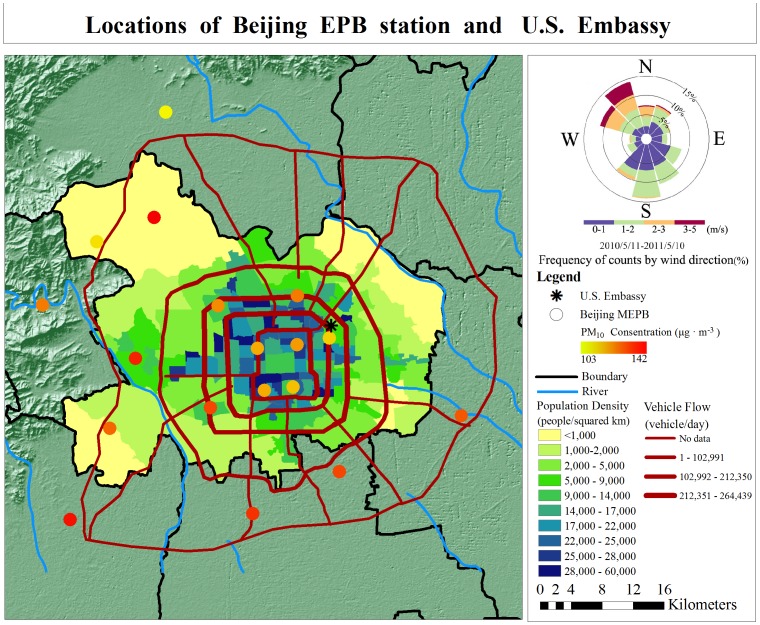
Location of BJ-EPB PM_10_ monitoring stations and U.S Embassy PM_2.5_ station (Beijing, China).

Daily PM_10_ concentration data were collected from May 10, 2010 to December 6, 2011 at the 18 authorized (BJ-EPB) observation stations, which are evenly distributed across the city. Daily PM_2.5_ concentrations reported by the embassy monitoring station were also gathered for the same period. Days with long periods of missing PM_2.5_ (hourly) data were discarded based on the following criterion: if during a day there were consecutive data gaps of more than 3 hours or the cumulative amount of missing data exceeded 12 hours, that day was not included in pollution estimation. The final result was a dataset covering a 423-day period. We also acquired information about the geographic locations of the U.S. Embassy and 18 BJ-EPB stations, as well as data on population density, main traffic routes, traffic flow volumes, daily mean wind direction and speed, and geomorphology. All data were stored in a Geographic Information System (GIS), and are represented in Figure 1.

### The SPA Technique

We developed a technique, called Single Point Areal Estimation (SPA), which belongs to the category of biased areal estimation techniques [23]. SPA was used to extend the temporal PM_2.5_ data recorded at a single (U.S. Embassy) monitoring station to areal-average PM_2.5_ pollutant estimates, taking advantage of physical correlations between the PM_2.5_ mass concentrations (U.S. Embassy station) and the PM_10_ data (18-station BJ-EPB network). This point-to-area transformation yields best linear unbiased estimates (BLUE) of PM_2.5_ spatial averages over the entire city of Beijing. A formal derivation of the SPA technique is given in the following.

**Figure 2:**
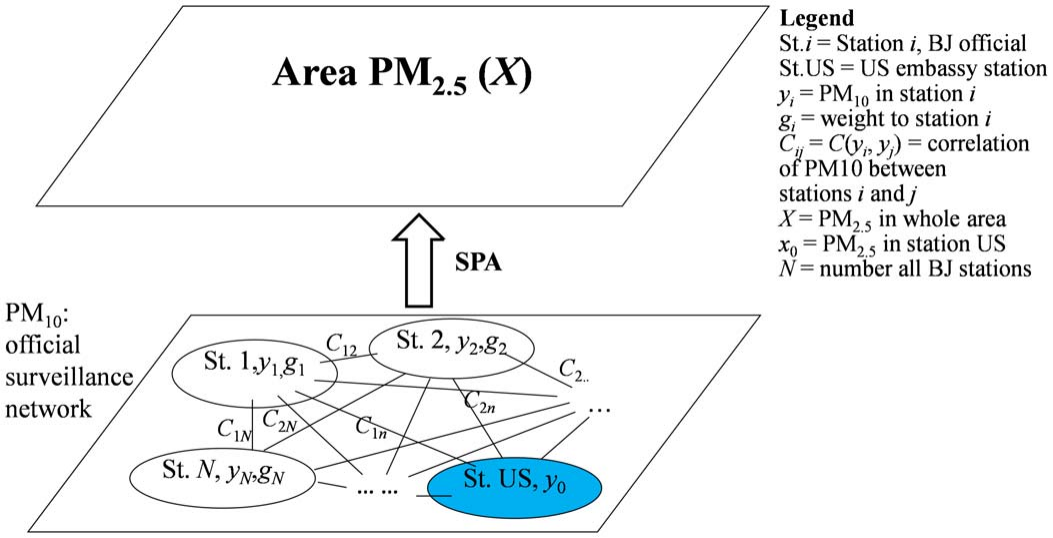
Relationship between stations and PM_2.5_ areal concentration: *y_i_* denotes PM_10_ concentration reported by station *i*, and *X* is areal PM_2.5_ concentration for Beijing; St. US denotes the U.S. Embassy station at which daily PM_2.5_ concentration x_0_ is observed; *X* is estimated by *x*_0_ using the SPA technique, based on observed PM_2.5_ data at the embassy station, and their correlation with PM_10_ concentrations observed at the 18 (evenly distributed) stations operated by BJ-EPB.

The objective of the SPA technique is to estimate citywide PM_2.5_ pollution in the Beijing area. The estimate is based on PM_2.5_ data from a single monitoring station at the U.S. Embassy in Beijing, and PM_10_ concentrations observations obtained at the official BJ-EPB monitoring network. Figure 2 outlines the SPA method.

The true average PM_2.5_ concentration (*X*) over the entire area per time unit (e.g., daily) is calculated in theory by
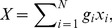
(1)where *x_i_* (*i = *1, …, 18) denotes PM_2.5_ concentration at the *i*-th station (which, in the present study, was not available from the official surveillance network); *N* denotes the total number of observation stations (18 in this case); *g_i_* denotes the weight (contribution) of the *i*-th observation station to PM_2.5_ estimation so that 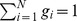 (unbiased estimation). There is only one PM_2.5_ monitoring station (U.S. Embassy). Accordingly, the areal PM_2.5_ concentration for Beijing is estimated by
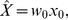
(2)where x0 denotes hourly PM2.5 concentration at the single monitoring station, as reported by the embassy and made available via the web site Twitter.com; w0 denotes the weight assigned to the embassy PM2.5 observation. This weight is estimated by minimizing
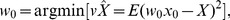
(3)where vX is the variance of the estimated area-averaged X ( = PM2.5 concentration); and the E(·) denotes statistical mean.

At the same time, it is valid that
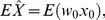
(4)i.e., the SPA technique generates an unbiased pollutant estimate that is also the best (in the minimum mean squared estimation error sense).

### Derivation of the SPA Equations

The variance of 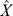
is derived as
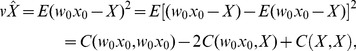
(5)where *C*(·) is the covariance between concentrations at any pair of points (the covariance provides a quantitative assessment of the spatial dependence between concentrations at these points).

The first term in Eq. (5) is
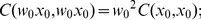
(6)the second term is
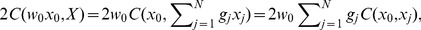
(7)and the third item is



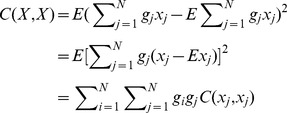
(8)By substituting Eqs. (6)–(8) into Eq. (5), we obtain
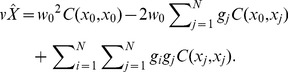
(9)


Taking into consideration the unbiased condition of Eq. (4), the Lagrange parameter *μ* is introduced into Eq. (9) in the following manner:
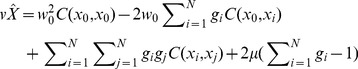
(10)


Minimization of Eq. (10) with respect to the 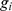
’s, 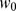 and 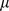 is a standard optimization problem, leading to the system of equations (to be solved with respect to 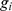, 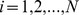, 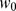 and 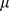):
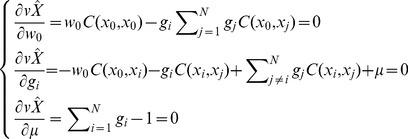
(11)


This system of equations can be written in matrix notation as
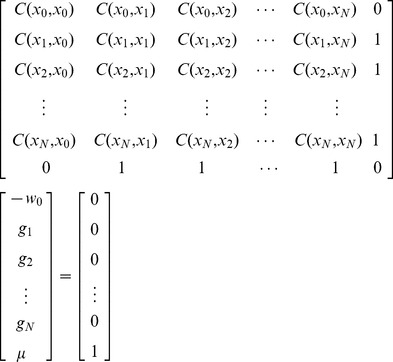
(12)


The solution of Eq. (12) yields *w*_0_, *g_i_* and *μ*, as appropriate.

### Accuracy of the SPA Technique

A variety of studies have discussed the uncertainty sources affecting the accuracy of data-based air quality estimates [24], [25]. Generally, there is an inverse relationship between uncertainty and accuracy – the higher the data uncertainty, the lower the accuracy of a model or technique. Usually the accuracy of a technique is measured in terms of its estimation error. The theoretical background of the **SPA** technique considers both horizontal correlations between samples, and vertical correlations between samples and area populations. It subsequently produces pollutant estimates that satisfy two key criteria – unbiasedness and minimum estimation error. Accordingly, **SPA** is a network-based estimation technique that is resistant to shifts [26] such as dust storms, which are addressed by statistical autocorrelation parameters in the model.

In this study, the horizontal (spatial) correlation between PM_2.5_ concentrations is approximated by that between spatial PM_10_ concentrations. The estimation error of this approximation is small due to various reasons:

- The citywide PM_2.5_ concentration estimated by SPA is defined as the weighted spatial PM_2.5_ average from all 18 stations (for each station the weight was proportional to the associated Voronoi area). Note that spatial topology –which is a key determinant of horizontal (spatial) autocorrelation [27]– is identical for PM_2.5_ and PM_10_ [28].
- Both particulates vary in space and time, subject to the same weather conditions, providing a valuable determinant of horizontal correlation [29], [30]. Vertical correlations between PM_2.5_ and PM_10_ concentrations were calibrated in terms of the observed data.
- Empirical evidence has shown that PM_2.5_ and PM_10_ concentrations are highly correlated, with values as high as 0.85 and 0.97, respectively [31], [32].
- In the SPA technique, the correlation coefficients between PM_2.5_ and PM_10_ are calibrated by the data so that they can correct for potential discrepancies (see section 2 in the *SI* text). Historical data have shown high correlations between the U.S. Embassy PM_2.5_ concentrations and the 18 PM_10_ observation stations (Table 1). The maximum and minimum values of Pearson correlation efficient are 0.85 and 0.69, respectively.

**Table 1:** Pearson correlation coefficient between the U.S. Embassy PM2.5 concentration and 18 Beijing EPB PM_10_ concentrations.

BJ-EPB Station	*r*	BJ-EPB Station	*r*
Aotizhongxin	0.81	Longquanzhen	0.82
Changpingzhen	0.72	Nongzhanguan	0.83
Dongsi	0.83	Tiantan	0.82
Fengtaihuanyuan	0.85	Tongzhouzhen	0.79
Gucheng	0.81	Wanliu	0.81
Guanyuan	0.83	Wanshouxigong	0.84
Haidingbeibuxinqu	0.69	Yizhuangkaifaqu	0.82
Huangcunzhen	0.80	Yungang	0.81
Liangxiang	0.82	Zhiwuyuan	0.77

Estimation precision was further assessed by a validation study using an exhaustive PM_10_ dataset in the study area. In particular, daily areal PM_10_ concentrations were estimated by the SPA technique based on records at each of the 18 PM_10_ stations. The actual daily areal PM_10_ concentration is the weighted spatial PM_10_ average from all 18 stations (for each station, the weight was proportional to the associated Voronoi area; see *Supporting material*). Subsequently, the areal PM_10_ concentration estimated by each of the 18 PM_10_ monitoring stations and SPA was compared to the actual concentration value, resulting in good agreement (Table 2 and Figure S1 in SI text). This result supports the reliability of the SPA technique when used to estimate areal pollution concentration based on a single monitoring station. An SPA software is provided that can be used to perform the data calculations of this study (www.sssampling.org/SPA). Readers can apply the SPA software to their own data.

**Table 2:** Summary of R^2^ values of the linear relationships between Beijing areal PM_10_ estimated on the basis of a single station using SPA and the true area.

BJ-EPB Station	R^2^	BJ-EPB Station	R^2^
Aotizhongxin	0.961	Longquanzhen	0.921
Changpingzhen	0.862	Nongzhanguan	0.966
Dongsi	0.969	Tiantan	0.941
Fengtaihuanyuan	0.961	Tongzhouzhen	0.867
Gucheng	0.933	Wanliu	0.947
Guanyuan	0.964	Wanshouxigong	0.971
Haidingbeibuxinqu	0.764	Yizhuangkaifaqu	0.888
Huangcunzhen	0.896	Yungang	0.925
Liangxiang	0.849	Zhiwuyuan	0.901

## Results

**Figure 3:**
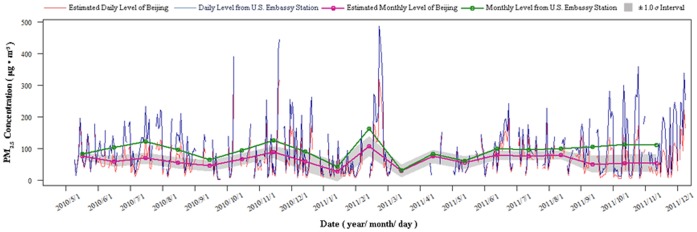
PM_2.5_ concentration observed by a single station (U.S. Embassy), and estimated citywide PM_2.5_ areal concentration (Beijing, China).

Daily PM_2.5_ mass concentrations observed at the embassy station ranged from 4 to 487 µg/m^3^ for the 423-day period. The annual average concentration (December 7, 2010–December 6, 2011) was 98.85 µg/m^3^, with high temporal variability. For the entire time series, the highest PM_2.5_ concentrations (>300 µg/m^3^) occurred during 10 days: December 7 and November 18–19, 2010, February 21–24, October 23 and December 5, 2011; see Figure 3.

During the same period, estimated citywide PM_2.5_ daily pollution in Beijing ranged from 2.86 to 318.29 µg/m^3^. The annual average pollution was 64.78 µg/m^3^. The highest concentrations (>300 µg/m^3^) occurred during two days, November 19, 2010 and February 21, 2011, as shown in Figure 3.

## Discussion

It was found that the U.S. Embassy PM_2.5_ observations exhibited approximately the same trend as citywide PM_2.5_ areal concentrations estimated by the SPA technique, although the embassy’s concentration values were clearly higher. The most important reason for this could be that the U.S. Embassy is at the city center, where population density and traffic volume are the highest in the city. The ratio between the embassy’s PM_2.5_ concentration and the estimated area-average concentration pollution varied with time. It is affected by the dynamic correlation between PM_2.5_ and PM_10_, caused by variation in local emission and atmospheric conditions between the embassy and the entire city.

Estimated area-average PM_2.5_ concentrations varied on a daily and monthly basis. The lowest concentrations occurred during January and March 2011, owing to the large number of windy days (refer to Figure S2 in *SI* text for monthly wind speeds). Estimation uncertainty is high for March 2011, because of serious data gaps. The highest concentrations occurred during July and November 2010, and during February and July–September 2011. During November, formation of a temperature inversion layer was observed over Beijing, which is a meteorological condition that plays an important role in the accumulation of PM_2.5_. The PM_2.5_ mass concentration peak during February was most likely due to emissions from coal consumption for heating purposes [33], [34]; this was the month with the lowest temperatures and slowest winds during 2011. July–September was the hottest period during a year. Long and intense solar irradiation during summer favors photochemical formation of aerosol particles [35], [36], which benefits the synthesis of PM_2.5_. This caused the high PM_2.5_ levels observed during that season. As regards seasonal variation, winter and summer had higher PM_2.5_ levels, with concentrations 68.74 µg/m^3^ and 70.42 µg/m^3^, respectively. Spring and fall concentrations were 63.59 µg/m^3^ and 61.54 µg/m^3^, respectively.

In sum, PM_2.5_ pollution in Beijing remained relatively high during the study period (Figure 3). Daily and annual interim target-1 standards recommended by the World Health Organization (WHO) are 75 µg/m^3^ and 35 µg/m^3^, respectively [37]. As mentioned earlier, the annual (December 7, 2010–December 6, 2011) average concentration in Beijing was 64.78 µg/m^3^. During that period, daily concentrations during 93 out of 259 days exceeded the WHO standard. Compared to the Beijing PM_2.5_ levels of five years ago reported in previous studies [33], [34], this level has dropped significantly. The situation may be attributed to a policy of prioritizing development of public transport, displacement of heavy industrial factories away from the city, and other efforts associated with the 2008 Beijing Olympics. Yet, the number of cars in the city has grown, from 2.6 million in 2005 to 5 million in 2010. Furthermore, air quality remains dependent on weather conditions, which means that considerable willingness and effort are needed to eliminate PM_2.5_ sources, thereby clearing the sky over the city.

### Conclusion

PM air pollution is a severe problem for Beijing city, as is demonstrated by both the official PM_10_ and the estimated PM_2.5_ concentrations. The areal PM_2.5_ concentration estimated by the proposed SPA technique was found to be a little lower than that observed at the U.S. Embassy monitoring station that is located at the city center and near a traffic junction. Validation results showed that the SPA technique is a useful tool in the estimation of areal PM2.5 concentration, even when only one PM2.5 observation station is available. Concerning the in situ implementation of SPA, (i) the key input to the technique is the correlation (covariance) between the PM_2.5_ and PM_10_ stations calculated from historical data, (ii) the estimation weight of the PM2.5 station was obtained by solving a linear equation (equation (12)) and, subsequently, (iii) the areal PM2.5 concentration was calculated from equation (2). Concluding, given the prohibitive costs of measurement campaigns and monitoring networks, the proposed SPA technique can be an effective and accurate pollution estimation tool, especially in cases in which, due to limited monitoring stations or in remote areas or in the past, other sources of information need to be used.

### Supporting Information

Details on data, estimation, the accuracy test, and a software of the method are available free of charge online at http://pubs.acs.org, or from www.sssampling.org/SPA.

## Supporting Information

Table S1 **Data description & model validation.**
(DOC)

Table S2 **Original data.**
(XLS)
