# Supplementary material for: Estimation of Citywide Air Pollution in Beijing
Source: PLoS One. 2013 Jan 8;8(1):e53400. doi: 10.1371/journal.pone.0053400 (PMC3539974; doi:10.1371/journal.pone.0053400)
Supplement: Table S1 — Data description & model validation. (DOC) [file pone.0053400.s001.doc]

Supporting Online Material for

**Estimation of Citywide Air Pollution in Beijing**

Jin-Feng Wang, Mao-Gui Hu, Cheng-Dong Xu, George Christakos, Yu Zhao

## Material

Beijing’s daily PM_2.5_ pollution level was estimated, based on the available PM_2.5_ and PM_10_ concentrations.

Daily records of PM_2.5_ concentrations were reported by the U.S. embassy’s observation station in Beijing, from May 10 (2010) to December 6 (2011). This hourly pollution report is available in the web site *Twitter.com* since spring 2008, and is based on a single monitoring station on the top of the embassy building. Daily PM_2.5_ concentration was estimated by averaging hourly concentrations. On certain days, there were missing data of hourly PM_2.5_. To maintain validity of estimated daily means, days with a substantial number of missing data were discarded using the following criterion: During a day, if there were consecutive data gaps of more than 3 hours, or the cumulative amount of missing data exceeded 12 hours, that day was not included in the pollution estimation. This resulted in a total of 423 days of valid data. Daily PM_2.5_ data are listed in Table S2.

Daily PM_10_ concentrations for Beijing were provided by official daily reports at 18 observation stations, which were published by the Beijing Municipal Environmental Protection Bureau (BJ-EPB). The data covered the same period as the U.S. embassy’s PM_2.5_ dataset. The daily PM_10_ data are displayed in Table S2.

## Method

- 1. **Validation of the SPA technique**

The validation of the SPA technique is guaranteed by the aforementioned theoretical derivations, and was empirically tested by means of an exhaustive PM_10_ dataset in the study area. The citywide PM_10_ pollution was estimated by SPA using the records of a single PM_10_ station of the BJ-EPB network. The actual daily PM_10_ is the area-weighted average of PM_10_ data from all 18 stations. The weight assigned to each station was calculated based on the proportion of Voronoi area within the total area. The areal PM_10_ estimated by each of the 18 PM_10_ stations using the SPA technique was compared to the true value, showing good agreement. This shows that the SPA technique can be reliable and should be used to estimate area values using only one monitoring station. Figure S1 and Table S1 show the linear relationships between PM_10_ pollution in Beijing estimated by the SPA technique and area-weighted averages.


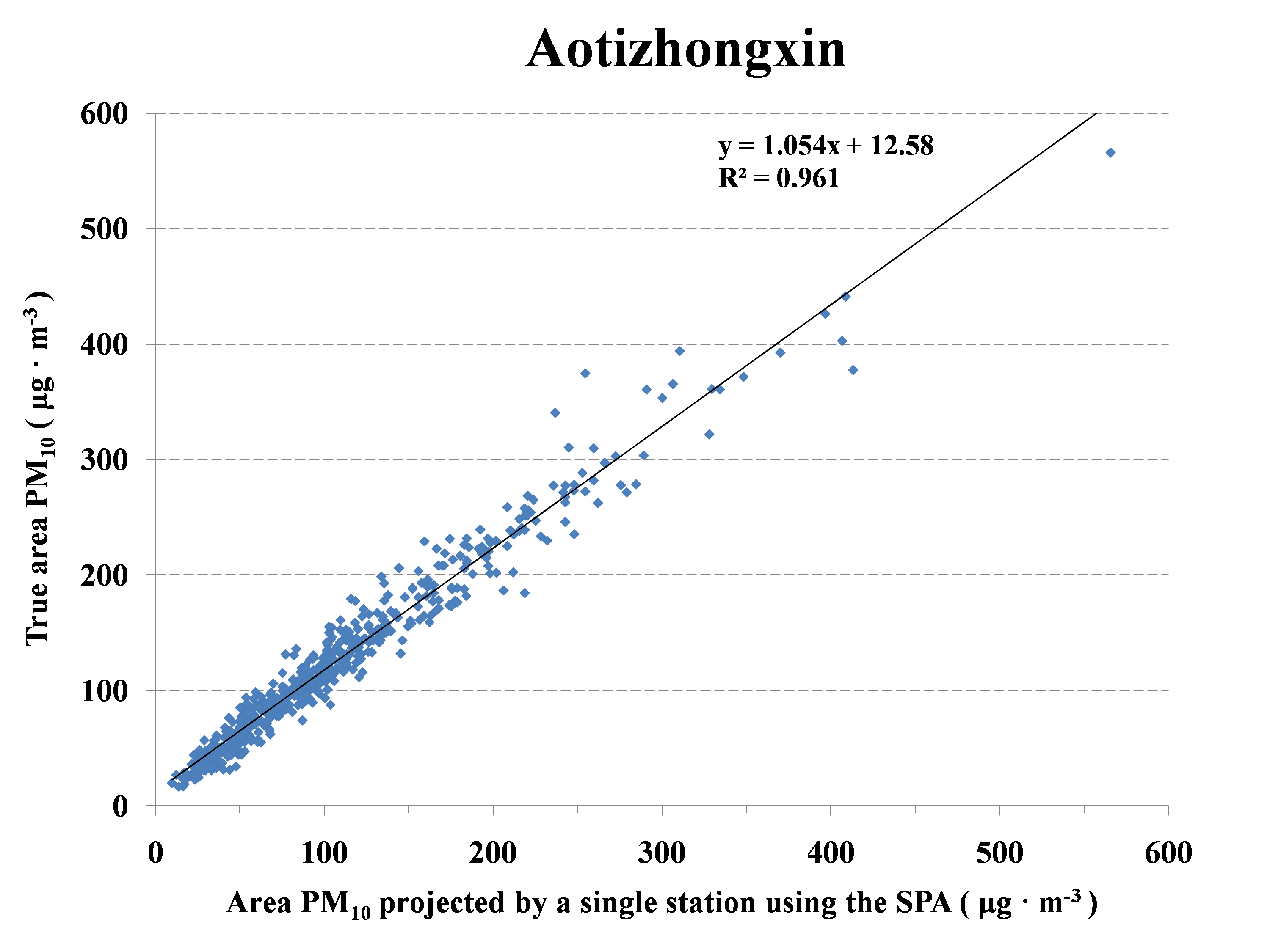

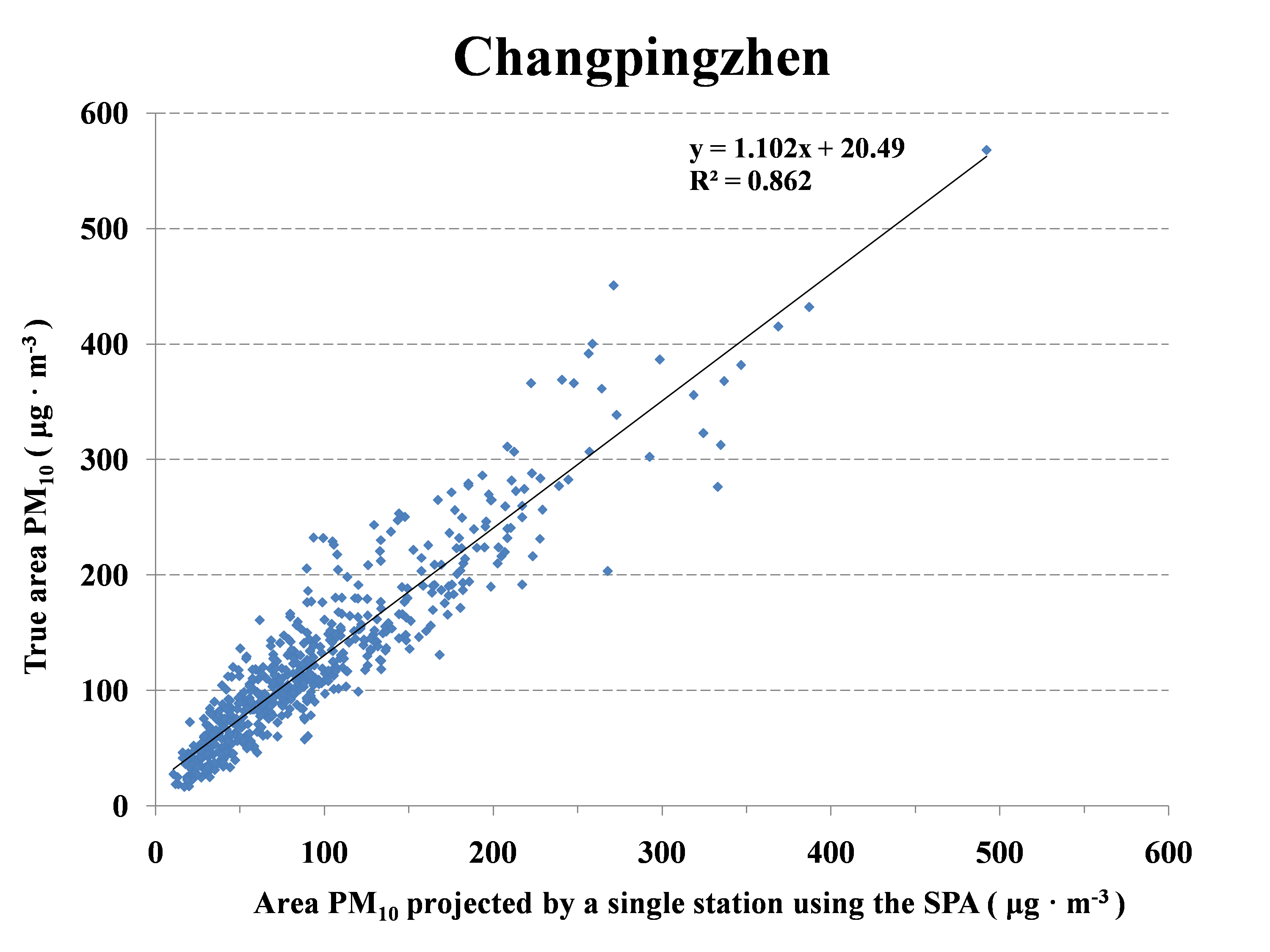

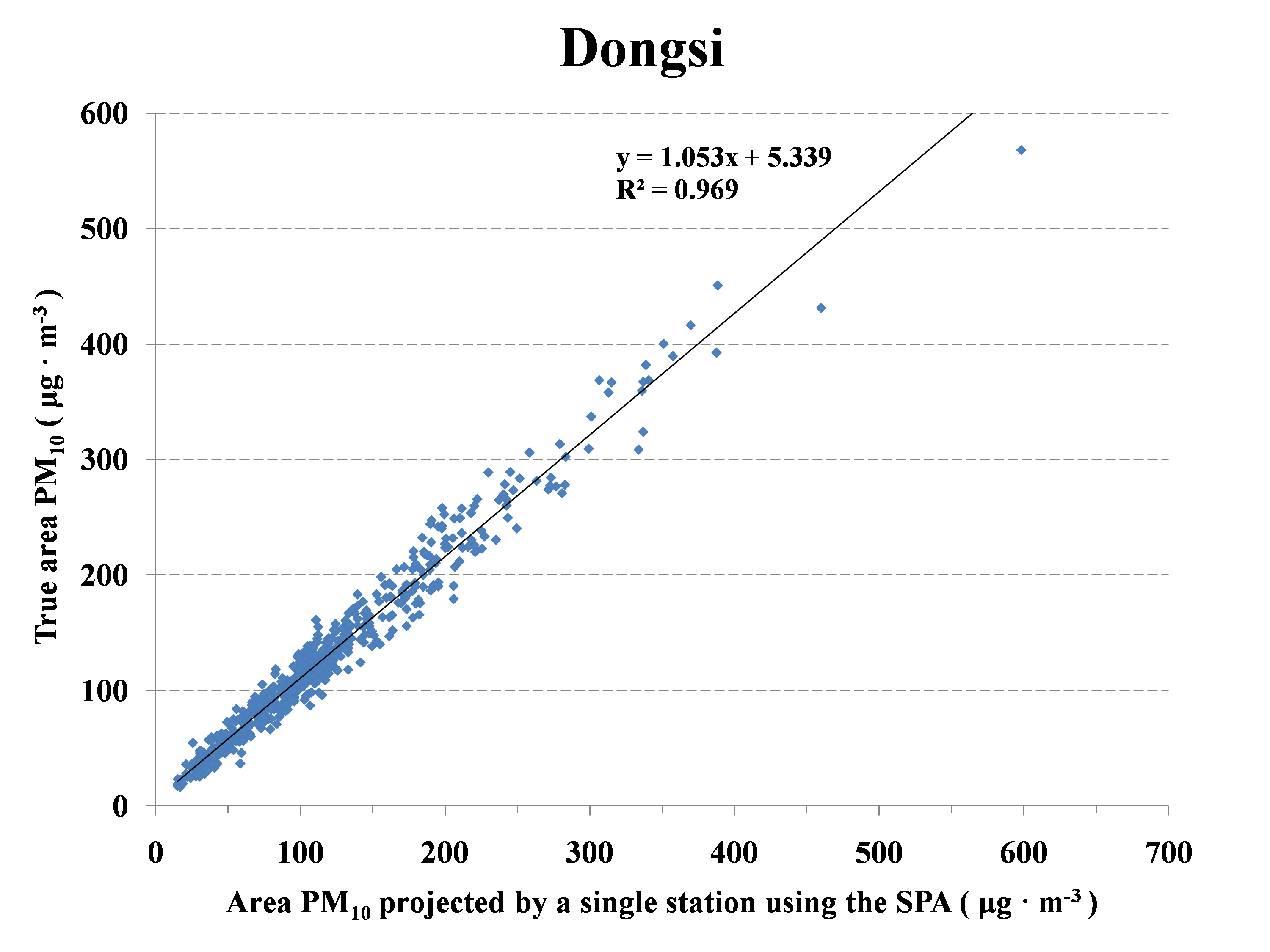

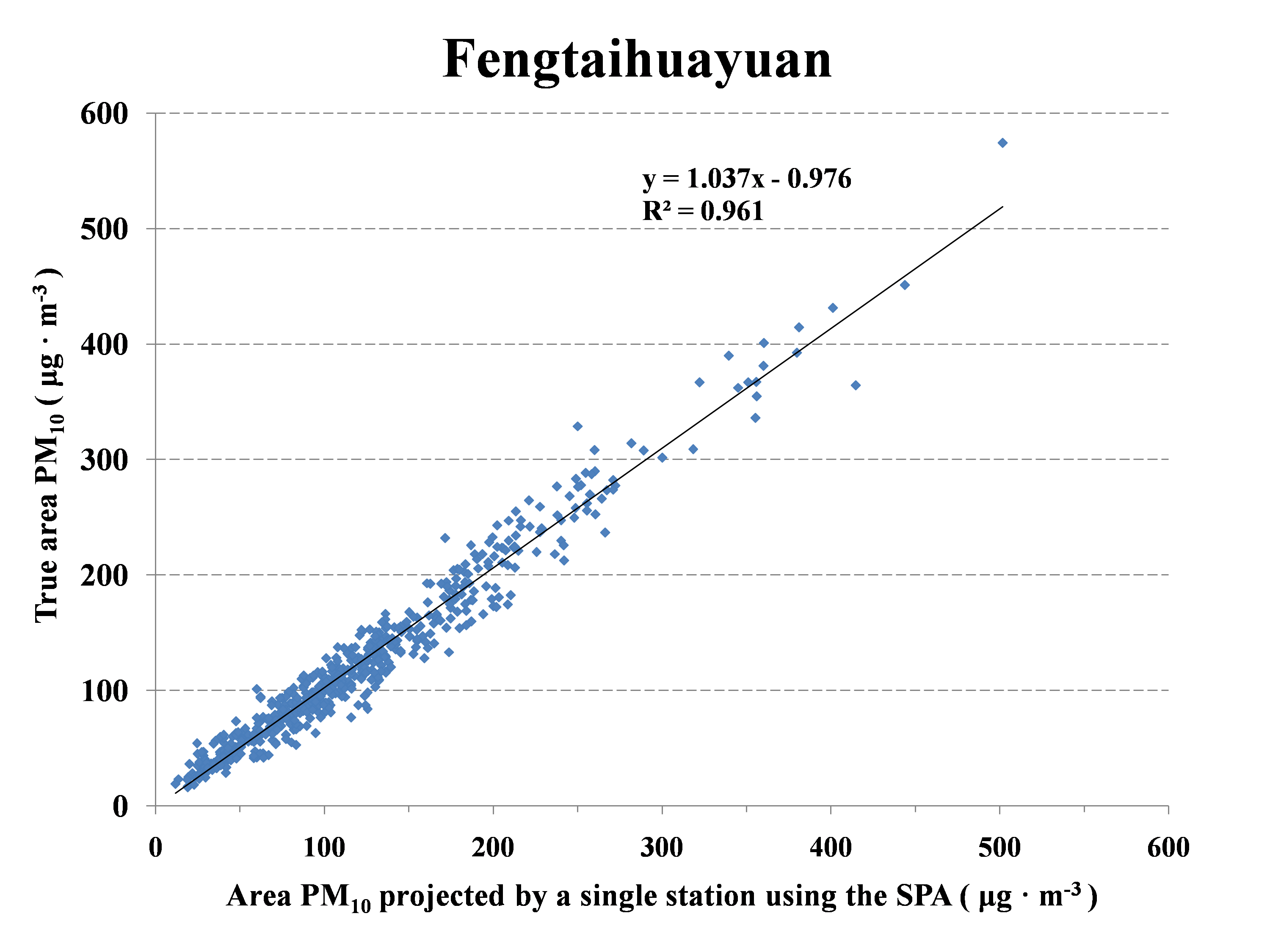

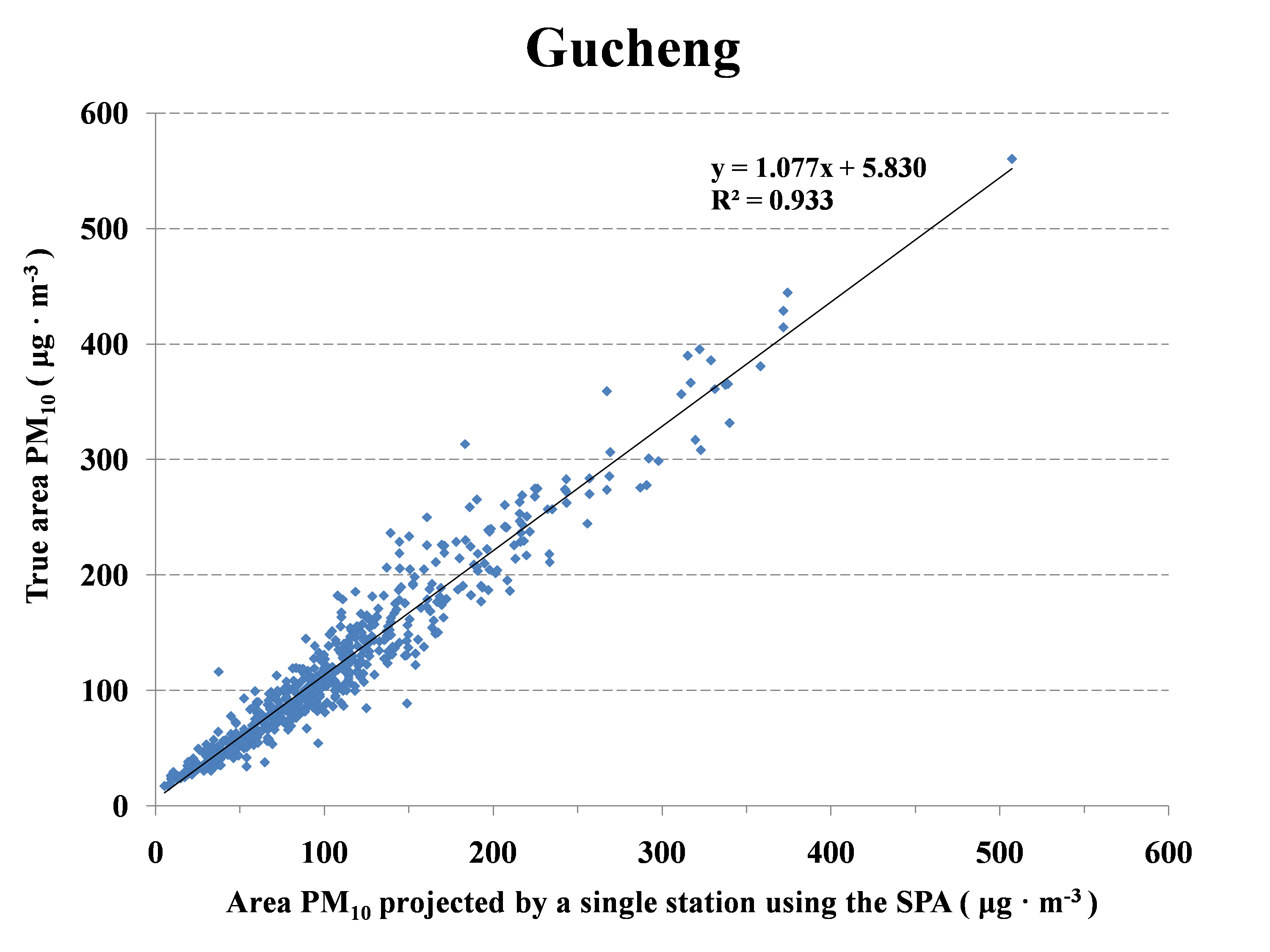

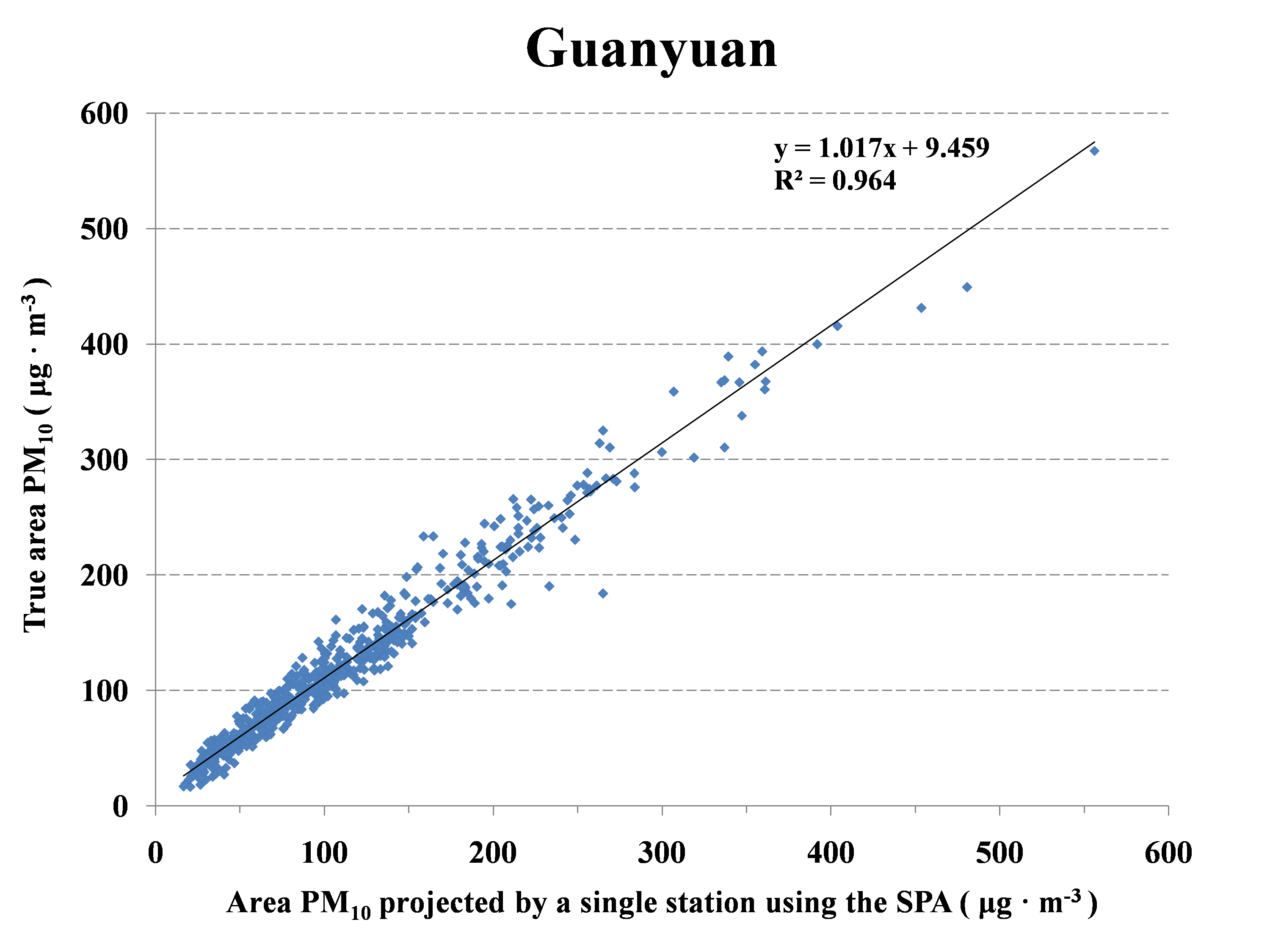

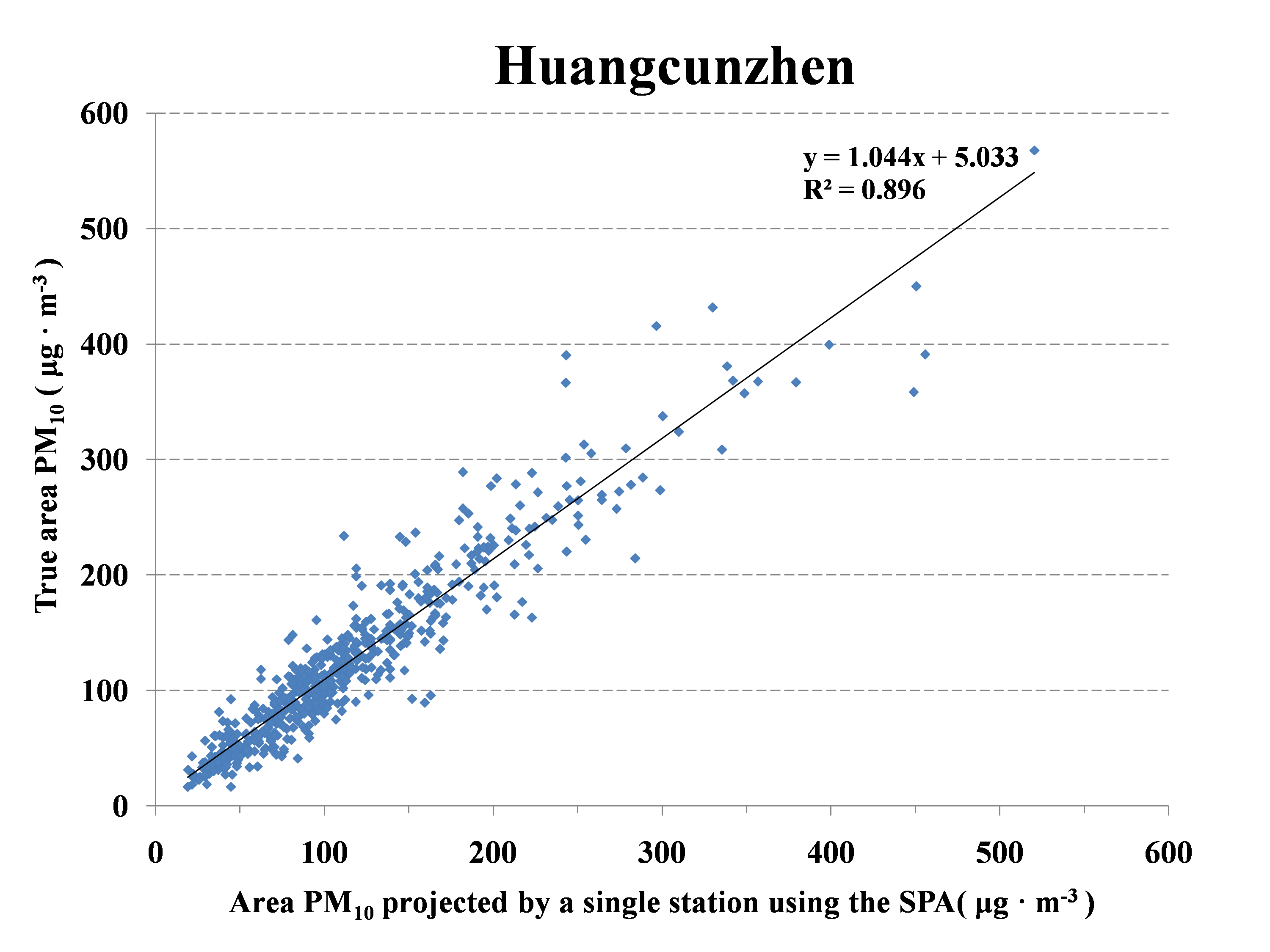

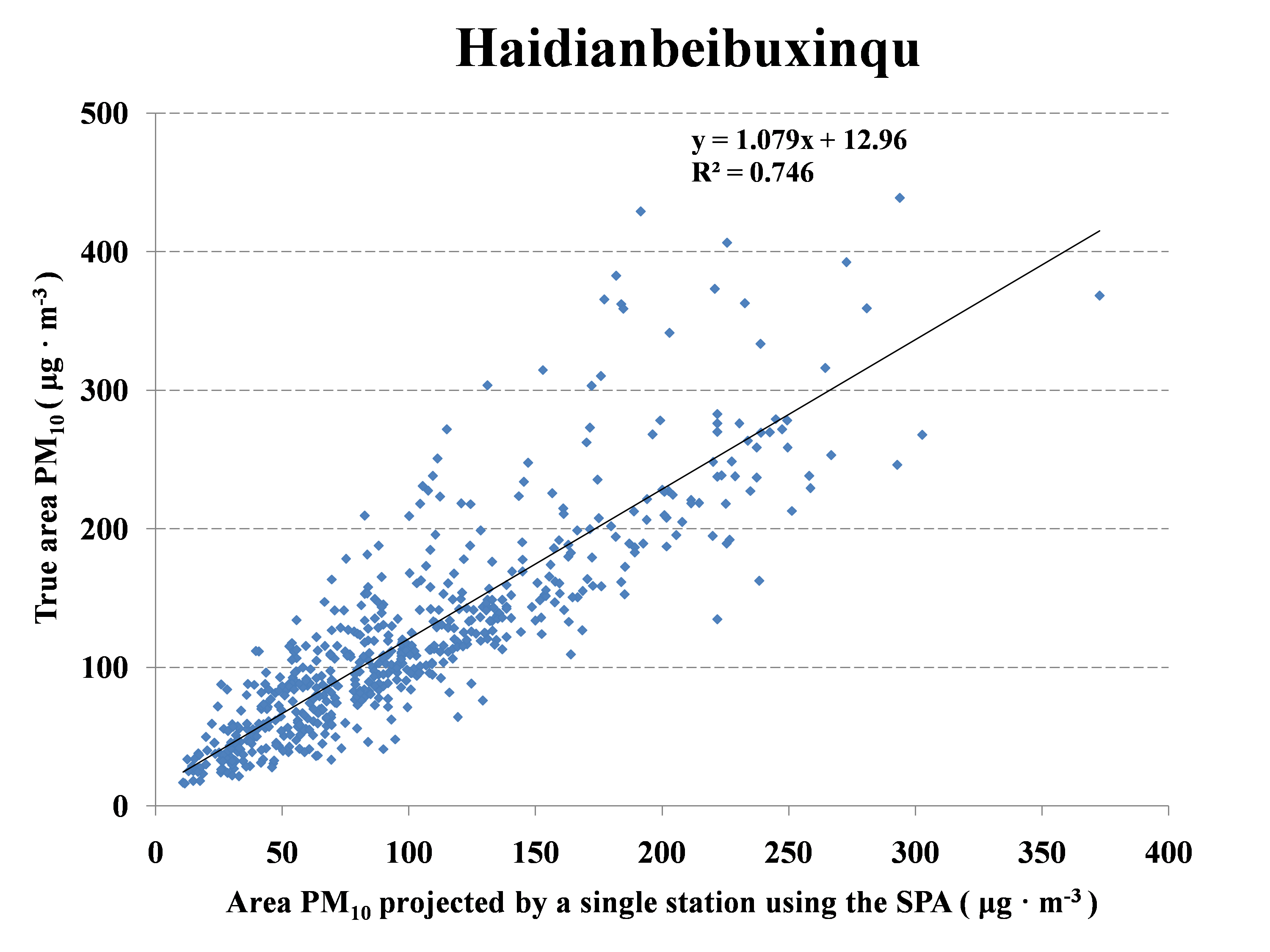

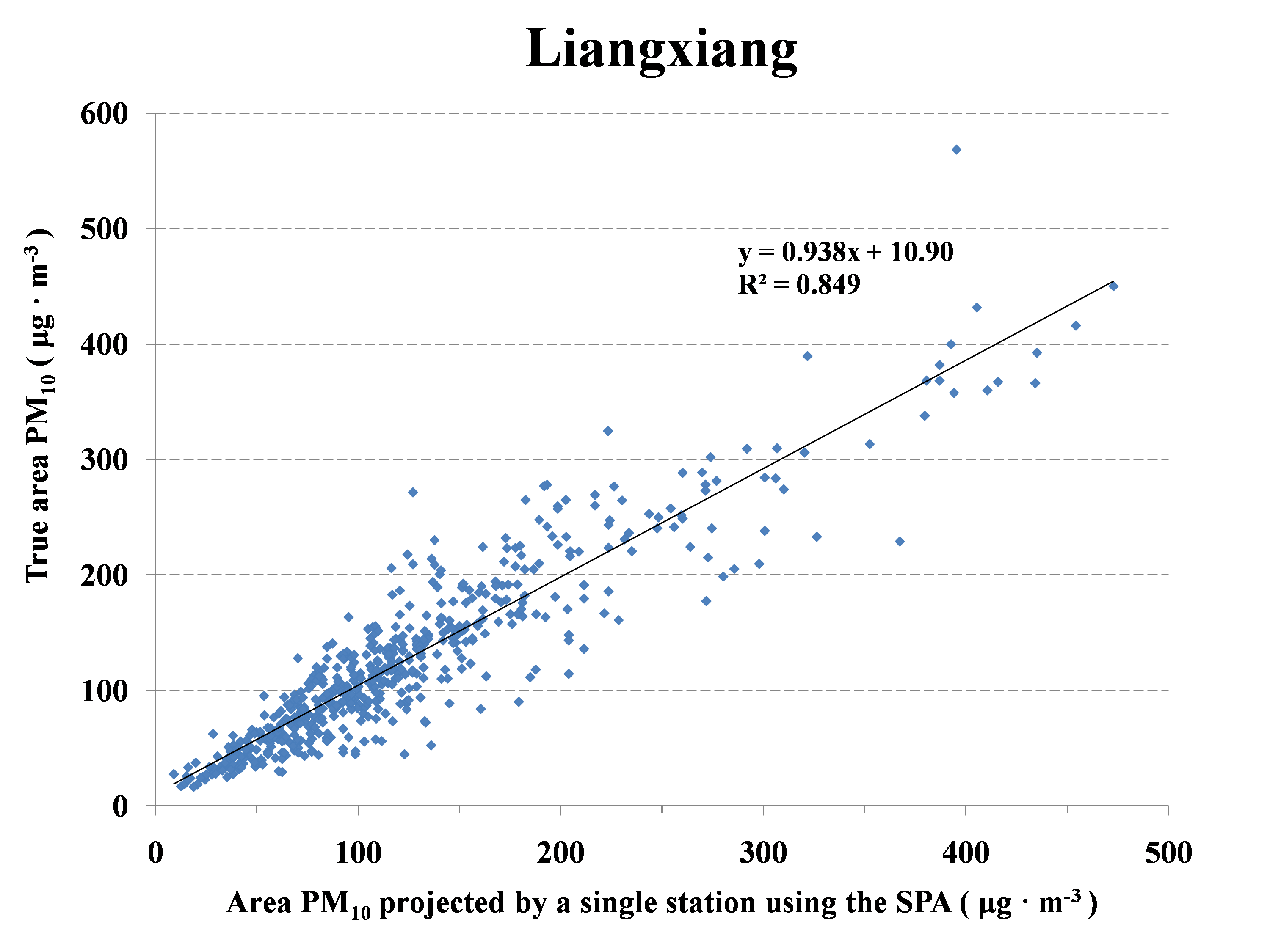

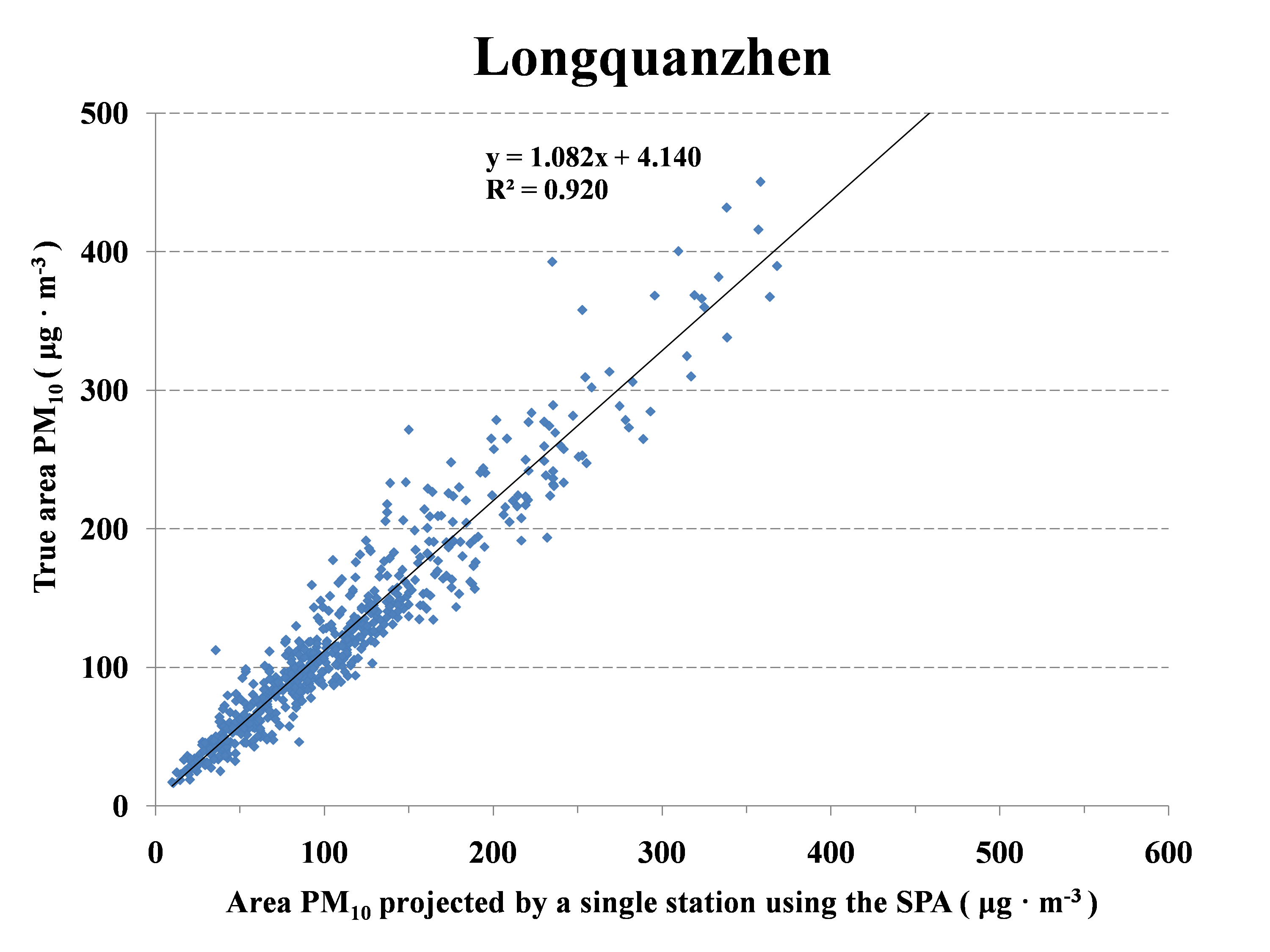

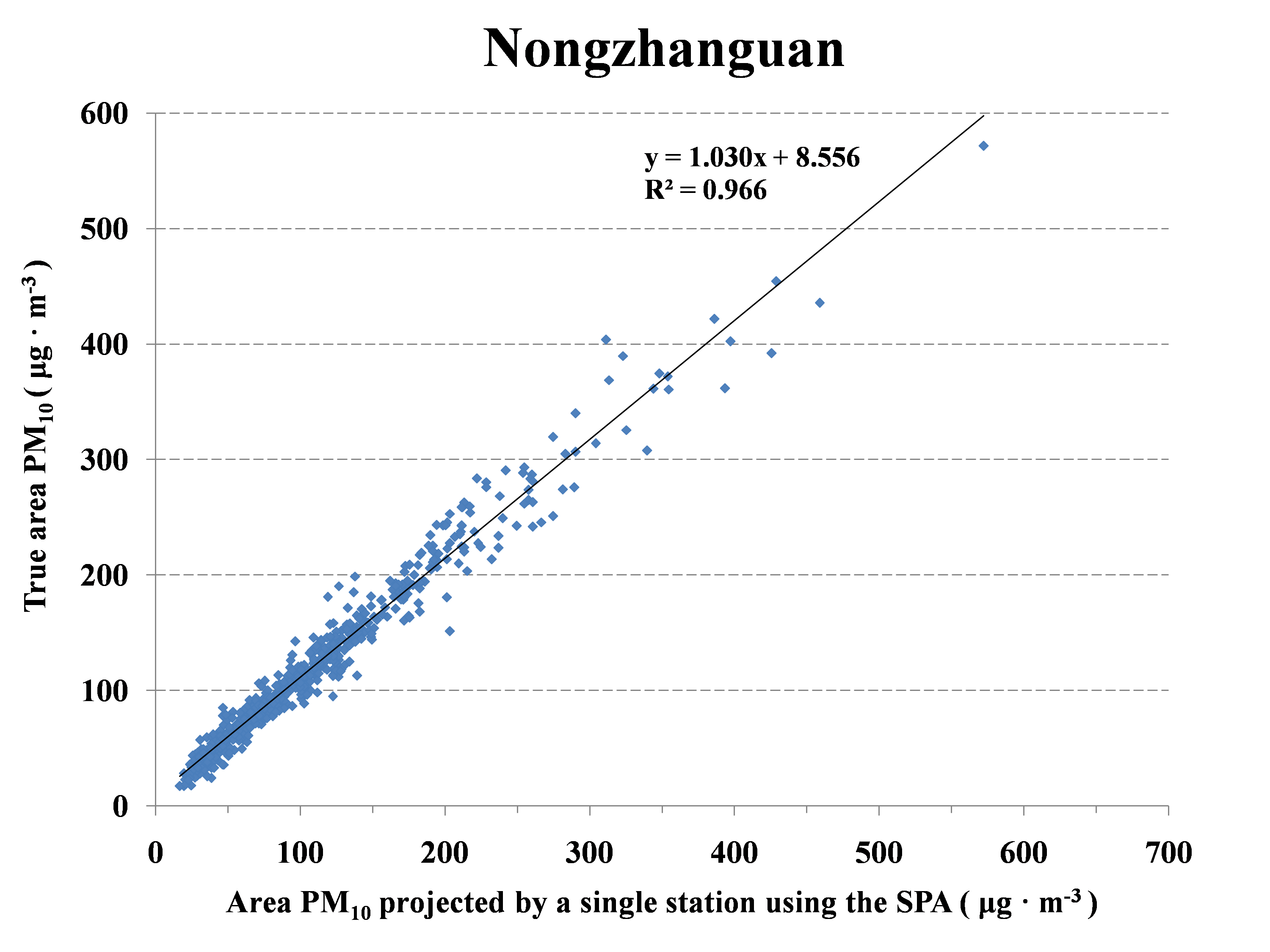

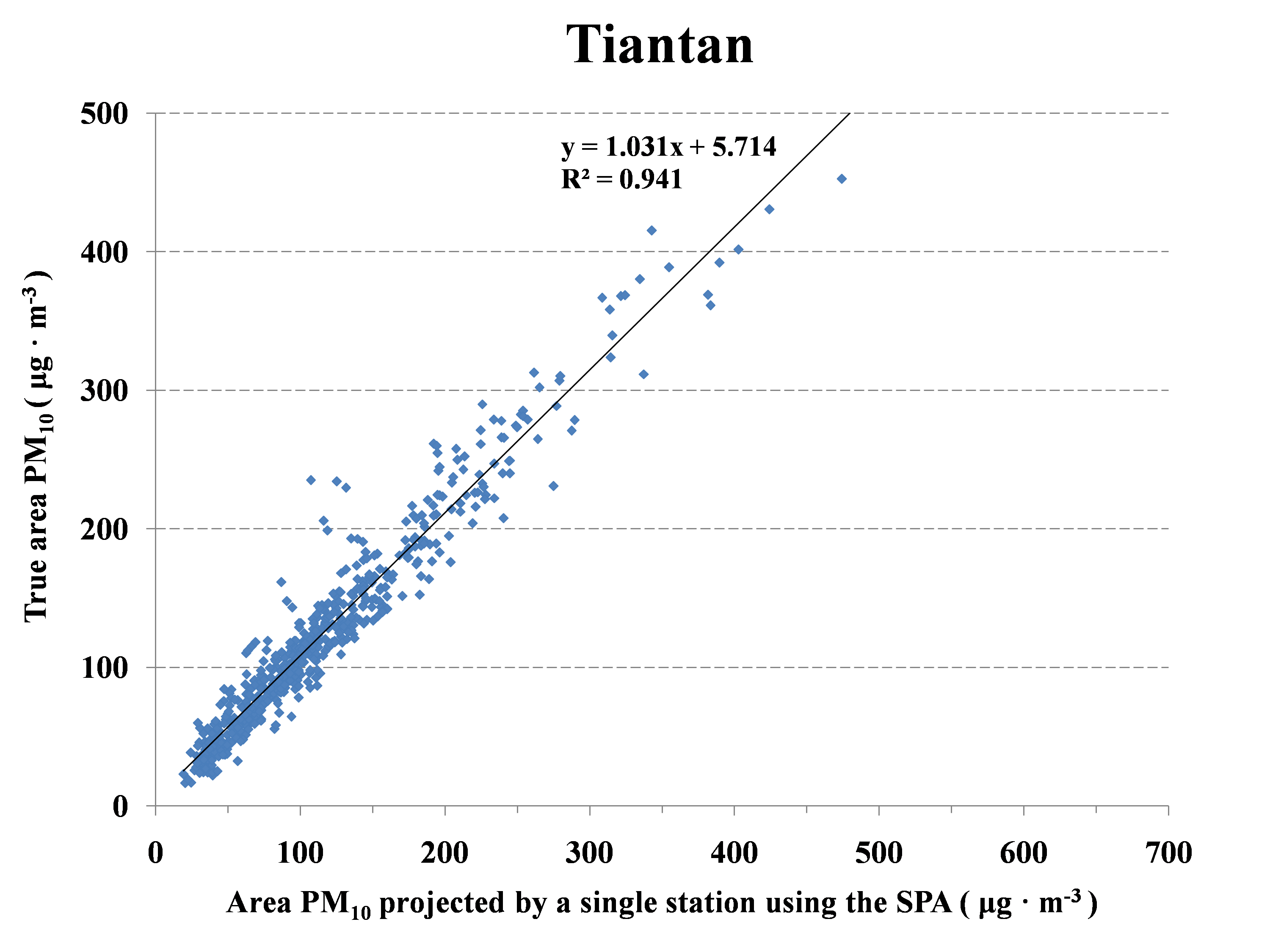

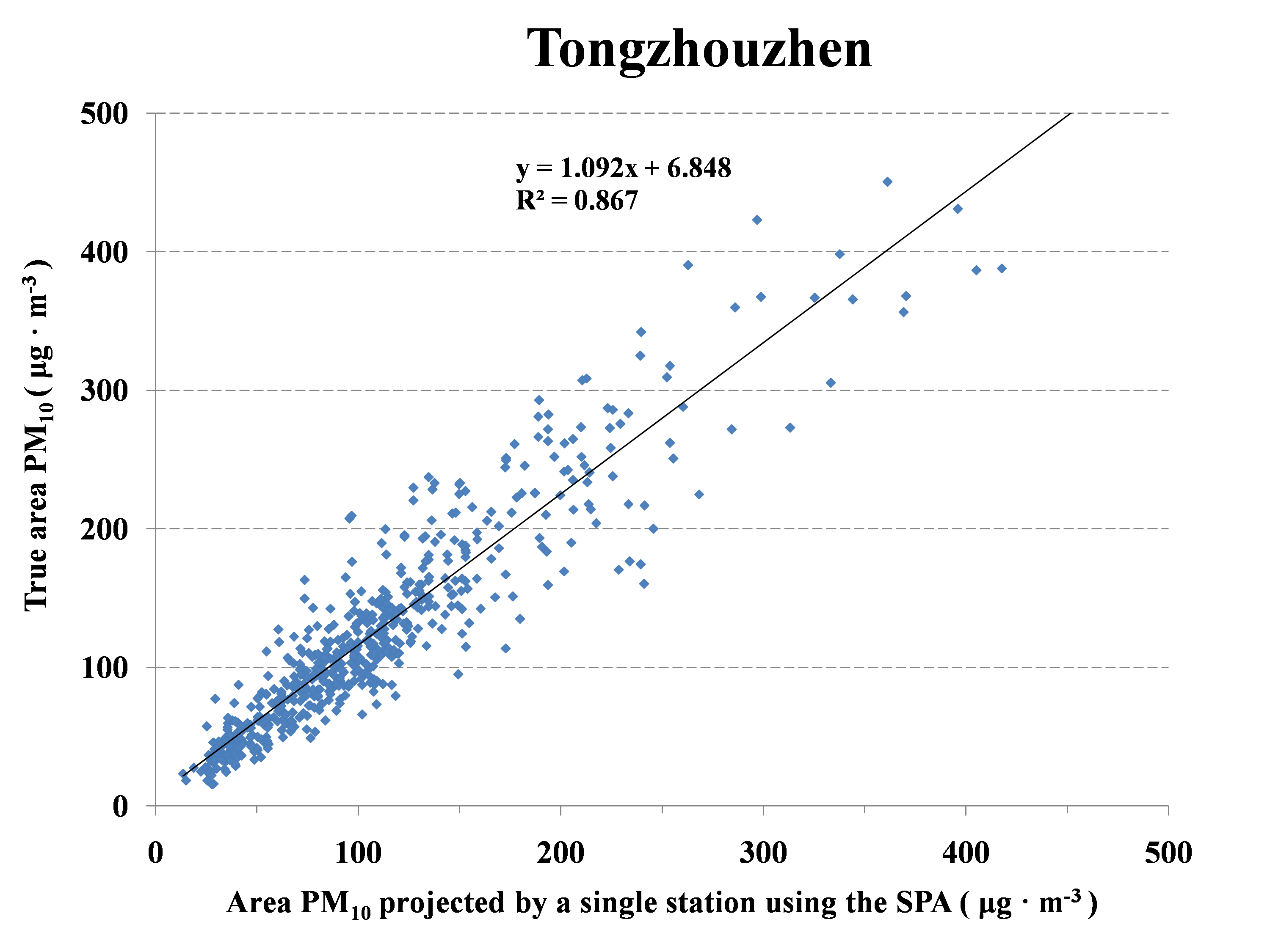

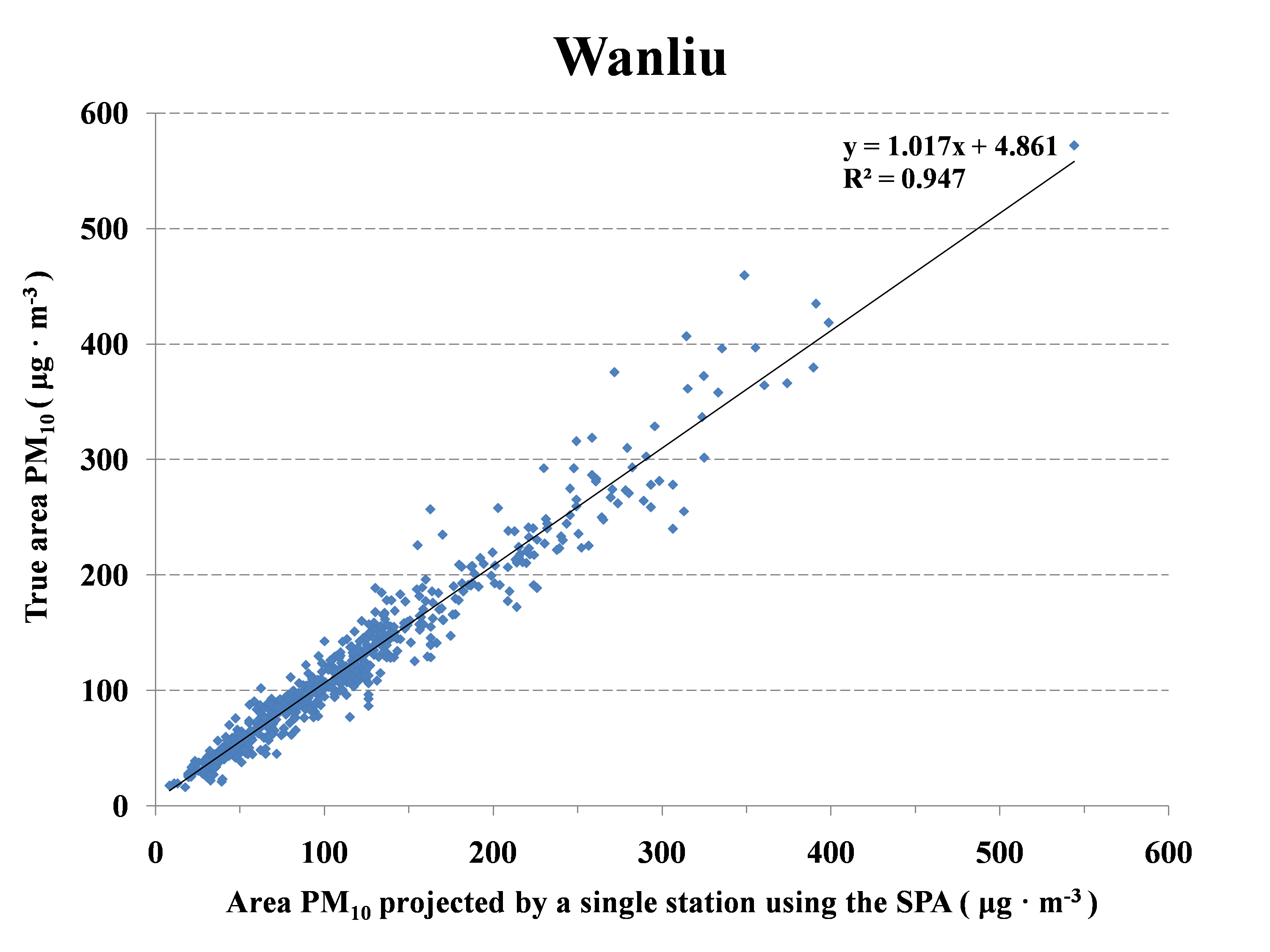

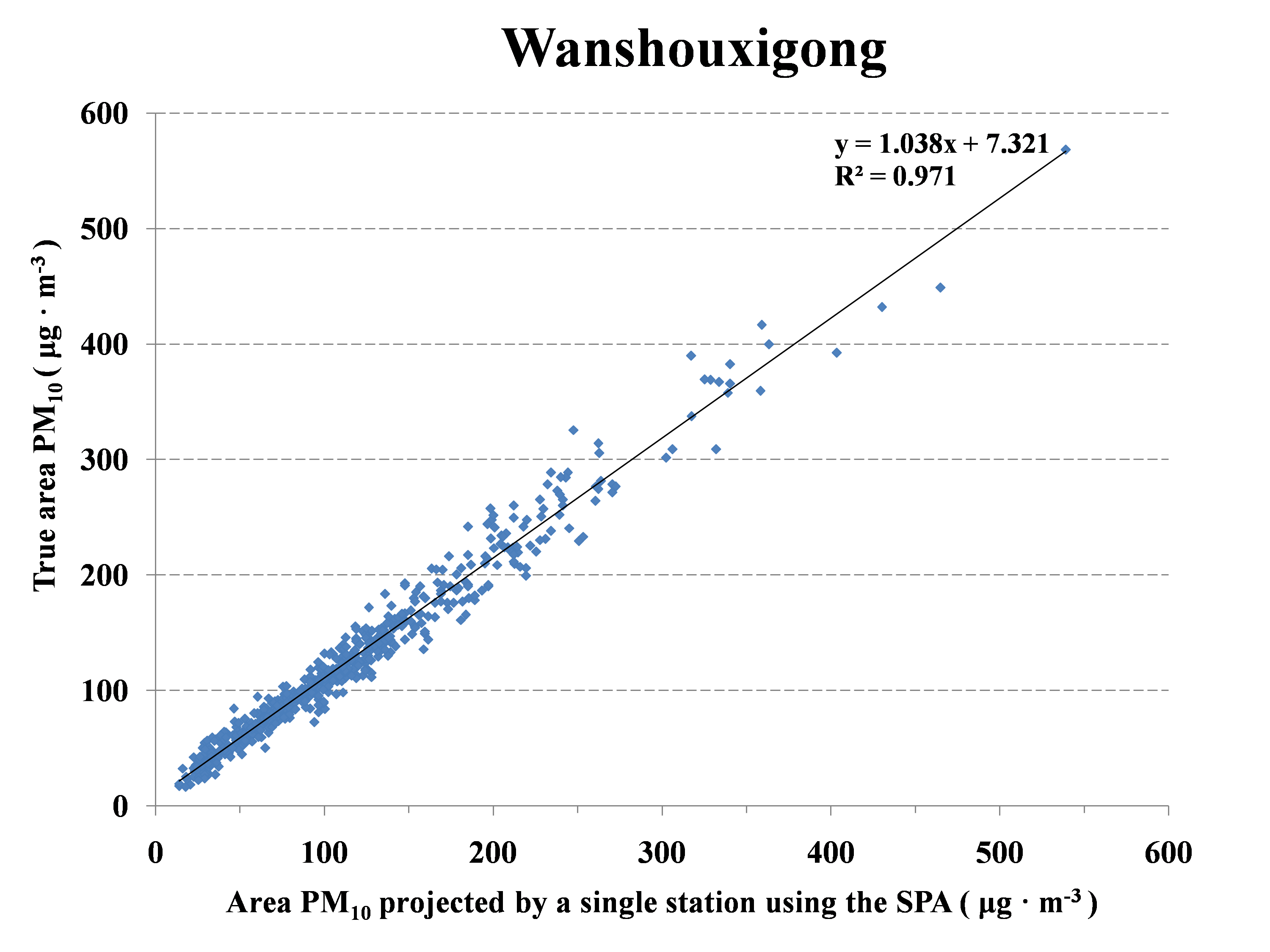

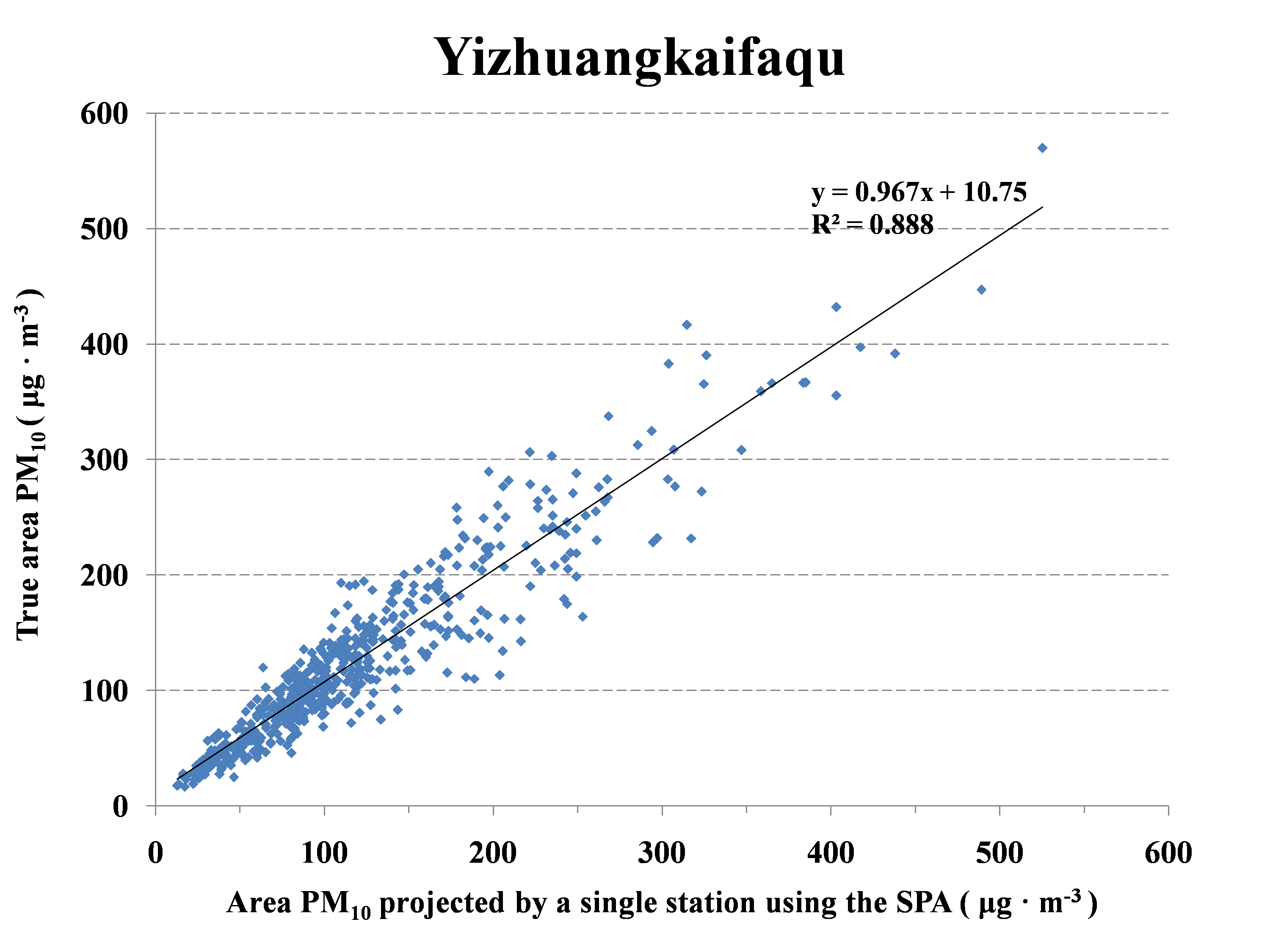

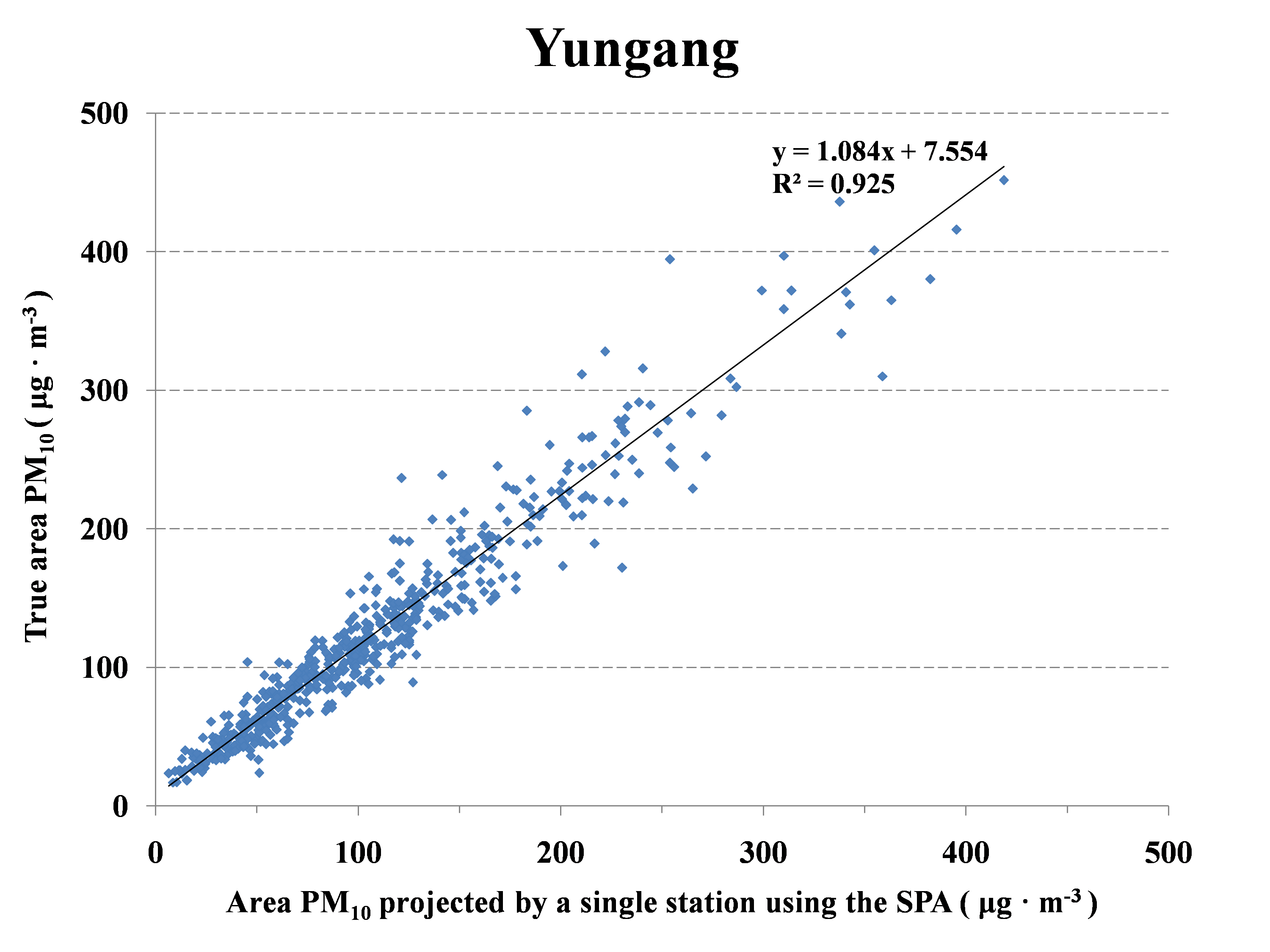

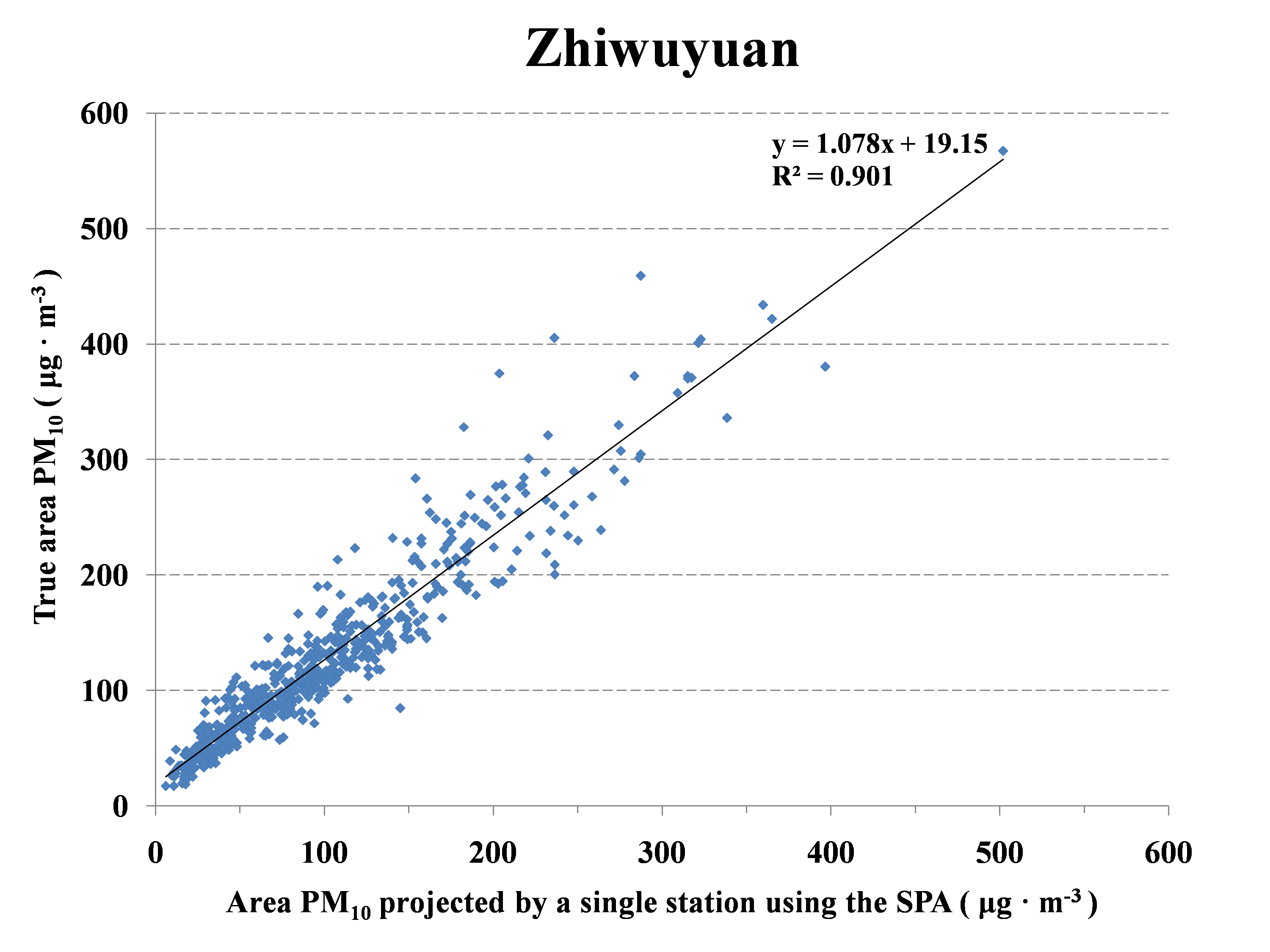


**Figure S1.** Linear relationships between Beijing area PM_10_ estimated on the basis of a single station using SPA and the true area concentrations.

**Table S1.** Summary of R^2^ values of the linear relationships between Beijing area PM_10_ estimated on the basis of a single station using SPA and the true area concentrations.

| **Beijing EPB Monitoring Station** | **R^2^** | **Beijing EPB Monitoring Station** | **R^2^** |
| --- | --- | --- | --- |
| Aotizhongxin | 0.961 | Longquanzhen | 0.92 |
| Changpingzhen | 0.862 | Nongzhanguan | 0.966 |
| Dongsi | 0.969 | Tiantan | 0.941 |
| Fengtaihuanyuan | 0.961 | Tongzhouzhen | 0.867 |
| Gucheng | 0.933 | Wanliu | 0.947 |
| Guanyuan | 0.964 | Wanshouxigong | 0.971 |
| Haidingbeibuxinqu | 0.764 | Yizhuangkaifaqu | 0.888 |
| Huangcunzhen | 0.896 | Yungang | 0.925 |
| Liangxiang | 0.849 | Zhiwuyuan | 0.901 |

- 1. **Estimation of Beijing PM_2.5_ pollution by the SPA technique**

The SPA technique was used to estimate the citywide PM_2.5_ pollution by integrating PM_2.5_ data at a single station with physical correlations between PM_2.5_ and PM_10_ observations available in Beijing municipal daily reports (Figure S1). The city-averaged PM_2.5_ concentration is expressed by Eq. (2). The weight (contribution) of the U.S. embassy PM_2.5_ monitoring station, *w*_0_, is calculated using Eq. (12). In particular, *,𝑥-0.* is the PM_2.5_ concentration observed at the embassy station, and the correlation between data *x_i_, x_j_* (*i* ,*j*= 1, 2, …,18) is calculated based on the daily PM_10_ values at the 18 official air quality stations, in which the correlation between PM_2.5_ values is approximated by that between PM_10_ values. This approximation, which is supported by physical data, may introduce some error, which, though, should be greatly reduced based on the following facts: i) Both PM_2.5_ and PM_10_ are (or assumed to be) measured at the same 18 stations; ii) both are obtained during the same weather conditions; iii) empirical evidence shows that they are highly correlated; and iv) the correlation coefficients between PM_2.5_ and PM_10_ in SPA is calibrated by the data, so they correct for potential discrepancies.


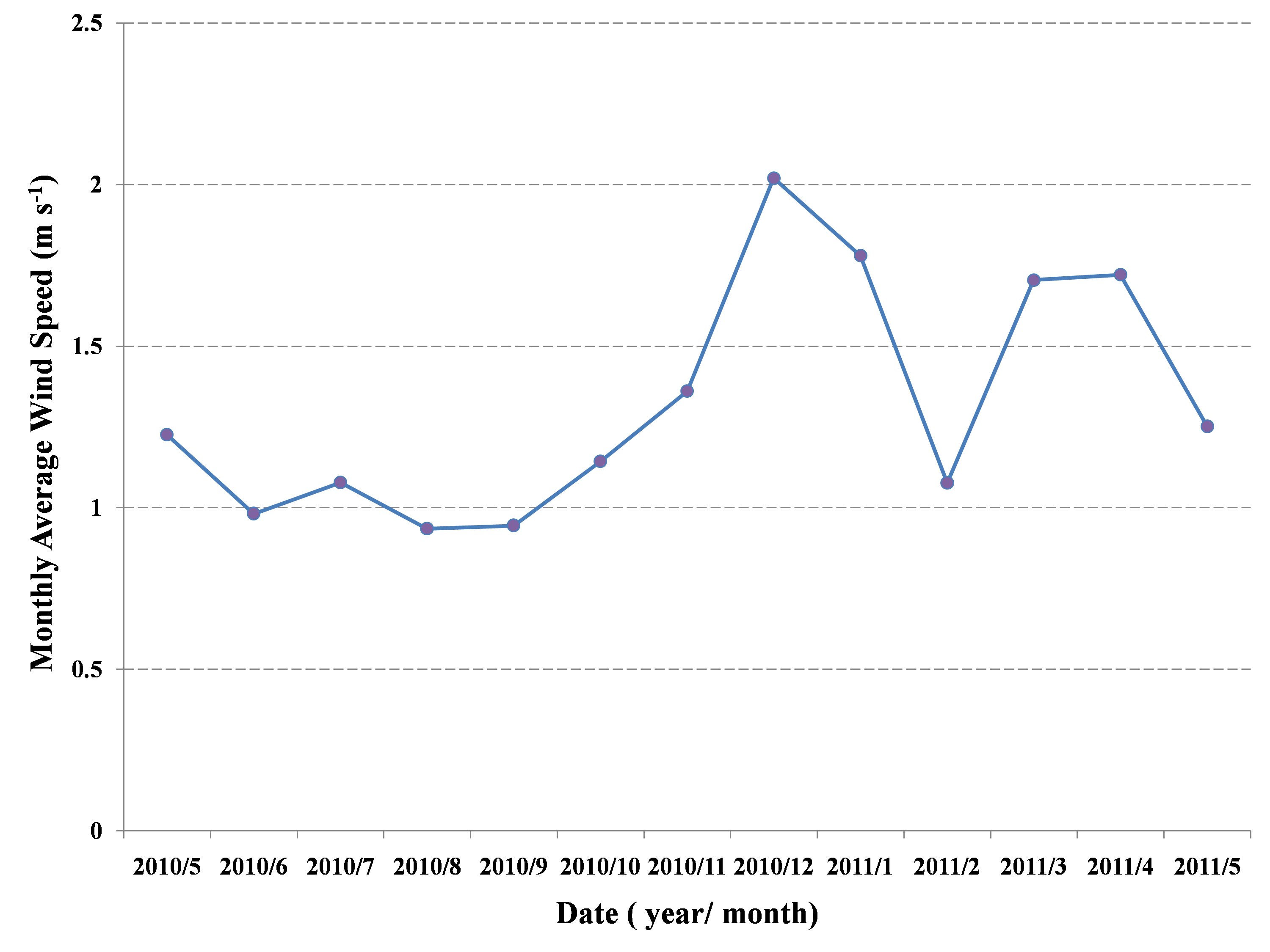


**Figure S2.** Average monthly wind speed in the Beijing area during the period May 2010 to May 2011
